# Three-Dimensional Reconstruction Based on Multiple Views of Structured Light Projectors and Point Cloud Registration Noise Removal for Fusion

**DOI:** 10.3390/s23218675

**Published:** 2023-10-24

**Authors:** Yun Feng, Rongyu Wu, Xiaojun Liu, Liangzhou Chen

**Affiliations:** 1 School of Mechanical Science and Engineering, Huazhong University of Science and Technology, Wuhan 430074, China; 2 Guilin Measuring & Cutting Tool Co., Ltd., Guilin 541000, China

**Keywords:** three-dimensional reconstruction, point cloud registration, structured light

## Abstract

Structured light technology is typical for capturing 3D point cloud data. This paper proposes a 3D reconstruction system to obtain point cloud data of complex objects based on nine-order Gray code and an eight-step structured light projection combined with a phase shift and phase unwrapping method. In this system, two projectors serve as bilateral projectors for structured light, along with a camera and rotating platforms. These components were used to obtain point cloud data from multiple perspectives, which helps avoid the shadow areas caused by a single projection angle and provides complementary point cloud data. The point clusters scanned under each perspective were transformed into the same coordinate system. Furthermore, a distance-based point cloud noise removal algorithm was proposed to optimize platform noise and facilitate point cloud data fusion. The experimental results proved that the system effectively captures 3D point cloud data for complex objects. The dimensional quantitative analysis of an aero engine blade was also performed.

## 1. Introduction

Three-dimensional reconstruction is a process used to obtain the surface shape of natural scenes, and it is widely applied in various fields, including weapons and equipment, industrial manufacturing, and more [1,2,3]. With the gradual popularization of related technologies, more and more 3D scanning measurement technologies based on planar structured light have been widely studied and applied due to their non-contact and high efficiency [4,5]. The principle of planar structure light 3D scanning measurement technology is to project a series of sinusoidal grating fringes with different phase displacements onto the object’s surface to be measured. At the same time, the camera is used to collect the deformed grating fringes modulated by the surface shape of the object to calculate the 3D topography data of the surface of the object to be measured through the process of phase solution and expansion [6]. Bergmann [7] first proposed in 1995 that the gray-coded grating-assisted phase shift method could effectively solve the ambiguity of periodic signals and obtain a higher spatial resolution. However, it required the encoded grating to correspond strictly with the phase grating. Otherwise, it would easily cause periodic dislocation and affect the phase accuracy of the solution. Guehring [8] proposed a method combining Gray code and line shift method, using a Gray code to mark the position of multi-line grating to eliminate the ambiguity between multiple lines. However, the Gray code boundary hopping can easily cause decoding errors, which leads to measurement errors. Song, Z. et al. [9] proposed the fringe movement technology, that is, the black and white binary fringe edges with the exact width of the code word as the Gray code word are used to mark the height information of the object and the binary fringe is moved three times within a cycle, each time by moving one pixel to mark each sub-area, to maintain the robustness in the measurement of high-reflection scenes. Chen [10] solved the phase information of space points in the image more accurately by integrating Gray code and six-step phase shift method encoding, providing a reliable calculation basis for later three-dimensional measurement and scanning. Wu [11] proposed a high-speed three-dimensional shape measurement method based on shifting Gray code light. The average intensity of the three phase-shifting fringe images captured was taken as the gray threshold to binarize the Gray code, thus eliminating the phase unwrapping errors caused by factors such as uneven reflectance, ambient light transformation, and projector defocus. Based on the shifting Gray-code encoding strategy, the residual error of phase unwrapping of the codeword edge is avoided. The above research is all based on the monocular structured light system, which uses the triangular relationship established by the single camera and projector to realize the three-dimensional reconstruction of the object. However, the monocular structured light system has the problem of shadow occlusion and cannot obtain the three-dimensional point cloud data of the measured object completely.

A binocular structured light system is proposed to obtain the measured object’s complete three-dimensional point cloud data. The binocular stereo vision bionic eye binocular parallax principle employs two identical cameras within a particular range at two different angles to simultaneously obtain images of the measured object. By calculating the position deviation in the two images in space, it can obtain the depth information of the measured object. Zhang Guangjun [12] designed a set of binocular structured light systems, which used two cameras and a projector to match the corresponding points of two images, overcoming the shortcoming of the binocular stereoscopic vision method, which had less information about the feature points on the surface of the measured object. Sharma [13] proposed a method for detecting cylindric-less metric information in stereo vision. The information is obtained by considering the isometry of the relative affine structure, which depends on the point depth invariance. The relative affine structure is used to represent the camera transformation of the cylindrical object to realize the measurement. Zhong [14] proposed a dual-projector auto-focus technology for 3D contour measurement. He added an electronic focusing adjustable lens in front of the camera to realize the camera’s auto-focus. He triangulated the pre-calibrated dual projector and a single camera to realize 3D reconstruction. Ying Yu [15] used two projectors to calculate the decoupled projection mode by time-frequency multiplexing, which could reduce the scan shadow and achieve a higher signal-to-noise ratio. ChuFan Jiang [16] developed the system calibration framework for the structural light system of a dual projector and single camera and applied it to three-dimensional measurement. Meanwhile, he proposed the residual correction method based on the system error function and the data fusion method of the angle between the projection direction and the surface normal. The experimental results show that the dual-projector structured light system can effectively expand the measurement range of the single-projector system and further improve the measurement accuracy. Zhang Song [17] introduced a structured light measurement system with two cameras and systematically discussed the system calibration method and the principle of 3D data registration using an iterative nearest point algorithm and 3D data merging of holographic images. Woolford and Burnett [18] described a set of dual-projector systems with a fringe pattern strategy based on spatial reuse. However, this method needs to ensure that the fringe pattern projected by the two projectors is vertical enough, which makes the technology impractical. Petkovic [19] proposed a time-specific phase-shift selection method to effectively separate projection sinusoidal fringe patterns, making them independent of the number and location of the projectors.

This paper presented the following aspects of work. (1) Based on previous studies, a structured light measurement system based on double projection and a single camera is proposed. Compared with the current mature monocular imaging system, it can effectively remove the shadow area caused by the structure of the measured object. (2) A multi-angle shooting with a rotating platform is proposed, which combines the parameter distance denoising algorithm of the rotating platform to remove the background noise of the point cloud and the platform point cloud data, and realize the point cloud data fusion; (3) The uncertainty experiment of 3D reconstruction of structured light is carried out by using standard devices, and the measurement accuracy is proved.

In summary, the monocular structure light measurement system is insufficient in practice because of its shadow occlusion, limited measuring range, and low light flux. To overcome the above drawbacks, this paper proposes a structured light measurement system based on a double projector and a single camera to eliminate the shadow area, enhance the light flux, obtain the point cloud data through the projection method combined with Gray code and phase shift method, carry out the multi-angle shooting on the rotating platform, and combine the rotating platform parameter distance denoising algorithm to remove the point cloud background noise and platform point cloud data. Finally, multi-view fusion and 3D reconstruction of point cloud data are realized. The Section 2 of this paper introduces the relevant projection theory, the theoretical calculation basis of the rotating platform, and the multi-viewpoint cloud fusion. In the Section 3, relevant experiments are carried out on objects such as aero engine blades and teacups, and the measurement uncertainty is calculated. The Section 4 discusses and summarizes.

## 2. Principle

The double projection and single camera 3D reconstruction system can solve the shadow occlusion problem in the single projection system but cannot realize the 360° panoramic reconstruction of objects. Multi-view object 3D reconstruction based on a rotating platform is a simple and effective method to obtain the complete point cloud data of the object’s surface. The object to be measured was placed on the rotating platform, which rotated at a specific angle. This allowed part of the object’s surface to move into the camera’s field of view successively, ensuring the acquisition of three-dimensional points reflecting different perspectives of the object’s surface. Afterwards, registration was completed according to camera and rotating platform calibration, and 3D reconstruction was realized.

### 2.1. Gray Code Combined with the Phase Shift Method

Structured light 3D imaging technology is widely used in mechanical engineering, intelligent manufacturing, and other fields due to its high precision and high efficiency [20,21]. Three-dimensional imaging technology based on structured light illumination projects the structured light field to the surface of the object through the projection device, takes the deformed light field through the imaging device at another angle, and finally obtains the 3D morphology information of the object through the demodulation of the light field information [22]. Sansoni [23,24] proposed the measurement method of Gray code combined with phase shifting technology. Frank [25] proposed Gray code technology in 1940, which required that when coding a group of numbers, any two adjacent codes had only one binary digit different. As a kind of structured light, Gray code itself can be directly used to modulate the shape of the measured surface. It can also be used to assist the information acquisition and calculation of other resulting light. The calculation process of Gray code is shown in Formulas (1) and (2) [22,26].

(1) $$\begin{array}{c} V\left(x,y\right)=\sum_{i=1}^{m}GC_{i}\left(x,y\right)2^{m-i} \end{array}$$

(2) $$\begin{array}{c} K(x,y)=i(V(x,y)) \end{array}$$

where *m* represents the total amplitude of Gray code, i(.) is only used to find and calculate the known relationship between the decimal code word *V* and the decoding code word *K*.

Phase shifting technique [27,28] is used to accurately shift sinusoidal fringe uniformly N(N⩾3) times in a cycle and the phase of each shift. The sine function expression of the projection fringe is shown in Formula (3), where A(x,y) represents the background light intensity and B(x,y)/A(x,y) represents the fringe contrast. Formula (4) is used to calculate the truncated phase of the shape information of the object. The 3D measurement based on phase shift technology can obtain a higher spatial resolution, but due to the arctangent operation, the phase will be truncated in between π and presenting periodic distribution. Therefore, the Gray code pattern marks the phase order *K* with sub-truncation, and Formula (5) expands the truncation phase [22].

(3) $$\begin{array}{c} I_{n}\left(x,y\right)=A\left(x,y\right)+B\left(x,y\right)cos\left[\phi \left(x,y\right)+2\pi \left(n-1\right)/N\right],n=1,2\ldots N \end{array}$$

(4) $$\begin{array}{c} \phi \left(x,y\right)=\arctan\left[\frac{\sum_{n=1}^{N}I_{n}\left(x,y\right)\sin\left(2n\pi /N\right)}{\sum_{n=1}^{N}I_{n}\left(x,y\right)\cos\left(2n\pi /N\right)}\right] \end{array}$$

(5) $$\begin{array}{c} \Phi (x,y)=\phi (x,y)+2\pi k(x,y) \end{array}$$

The encoding method combining Gray code and phase shifting technology can obtain the phase information of space points more accurately and provide an accurate calculation basis for the late three-dimensional measurement. Therefore, this paper proposes a structured light projection technology based on a nine-order Gray code combined with eight-step phase shift technology and auxiliary projection of static all-black and all-white Gray code graph to determine the threshold value. Compared with the common monocular 3D reconstruction system and binocular 3D reconstruction system, this paper uses double projectors and cameras to constitute a 3D reconstruction system to achieve left and right supplementary projection, which can effectively avoid the phenomenon that the shadow cannot be reconstructed when the projection light is blocked by the protruding parts of the object to be measured.

**Figure 1:**
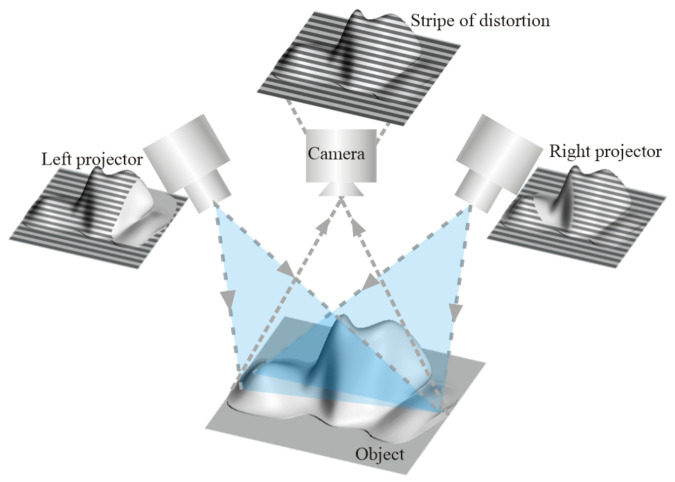
Dual projectors combined with a rotating platform can effectively remove the influence of shadow areas on the measurement.

As shown in Figure 1, when there are protruding edges and corners on the surface of the object to be measured, the projection of structural light by the right projector at a certain angle will be blocked by the protruding edges so that the structural light fringe cannot cover the shadow area on the surface of the object, resulting in the phenomenon of point cloud holes when the point cloud is formed. To overcome this problem, a three-dimensional reconstruction system composed of dual projectors combined with a single-phase mechanism is adopted. When the left projected light is blocked by the object, a point cloud hole is formed in the shaded area. The projected light of the right projector is not necessarily blocked by objects, and the point cloud data formed by the left and right projectors are intersected, combined with the point cloud data features generated by the left and right projectors, and part of the point cloud noise is removed. In the subsequent multi-view point cloud data fusion process, the fusion of the left and right point cloud data under different angles can realize the filling of point cloud holes.

### 2.2. Calculation of Rotation Matrix

In the existing 3D reconstruction system of structured light, the position of the measured object and the imaging device is relatively fixed so the measured object’s surface shape information can only be obtained from a single perspective. If a complete three-dimensional model of the object surface needs to be built, the measured object must be photographed and measured from multiple angles [29,30]. Since the measurement coordinates systems of local 3D point cloud data in a single shot differ, coordinate changes are needed to achieve a rough registration of point cloud data to unify the obtained 3D point cloud data into the same coordinate system.

**Figure 2:**
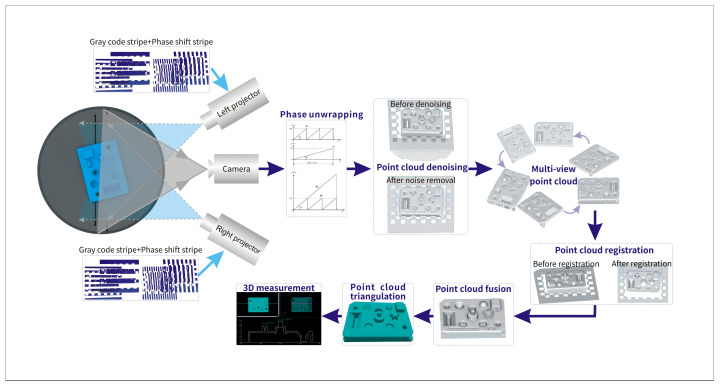
Schematic diagram of multi-view 3D reconstruction principle.

Before multi-view point cloud registration, it is necessary to calculate the rotating platform’s three-dimensional plane and rotation axis to eliminate the error point cloud. To this end, the center coordinates and axis equations of the circular rotating platform were calculated using a calibration checkerboard. According to the corresponding relationship between the projector and the camera, the three-dimensional coordinates of the object to be measured [27,31] are calculated using the principle of triangulation, as shown in Formula (6).

(6) $$\begin{array}{cc} s_{c}{\left[u_{c},v_{c},1\right]}^{T} & =A_{c}M_{c}{\left[X_{w},Y_{w},Z_{w},1\right]}^{T}\\ s_{p}{\left[u_{p},v_{p},1\right]}^{T} & =A_{p}M_{p}{\left[X_{w},Y_{w},Z_{w},1\right]}^{T} \end{array}$$

where sc and sp are respectively the scale factor of the camera and projector, (uc,vc) and (uc,vc) are the image coordinates of the camera and projector, both corrected by using the pre-calibrated system distortion parameters. Xw,Yw,Zw,sc,sp,up, and vp are unknown in the formula, and there are seven linearly independent equations in the two formulas, so the two formulas can uniquely determine the three-dimensional coordinates of the measured points, as shown in Figure 2.

The initial rotation platform coordinate system is placed in the camera coordinate system, and the relation between the initial turntable coordinate system and the rotated coordinate system can be obtained according to the rotation matrix transformation relation. The checkerboard picture used in camera calibration was used to calibrate the rotating platform, and the coordinates of the center of the circle and the equation of the rotating axis were calculated. The plane equation of the rotating platform obtained by the checkerboard corner point rotation is:

(7) $$\begin{array}{c} M\left(x-x_{0}\right)+N\left(y-y_{0}\right)+K\left(z-z_{0}\right)=0 \end{array}$$

Further, it can be simplified as: $\left(M,N,K\right){\left(x,y,z\right)}^{T}=n{\left(x,y,z\right)}^{T}=D$, where, n=(M,N,K) is the normal vector of the plane, (x,y,z) is the coordinates of any point on the rotating platform, $D=-n{\left(x_{0},y_{0},z_{0}\right)}^{T}$ is the plane coefficient, and $\left(x_{0},y_{0},z_{0}\right)$ is the coordinates of the center of the circle.

The rotation matrix of the point cloud of the measured object can be obtained according to the center and axis of the rotating platform. It is assumed that the center coordinates obtained by the calibration of the rotating platform are $\left(x_{0},y_{0},z_{0}\right)$, and the normal vector is $\left(n_{x},n_{y},n_{z}\right)$. When the rotation angle is 1°, let k=1−cos(π/180), $m=n_{x}+x_{0}+n_{y}y_{0}+n_{z}z$, The terms of the rotation matrix are:

$$\begin{array}{c} t_{00}=kn_{x}^{2}+\cos\left(\frac{\pi }{180}\right),t_{01}=kn_{x}n_{y}-n_{z}\sin\left(\frac{\pi }{180}\right),\\ t_{02}=kn_{x}n_{z}+n_{y}\sin\left(\frac{\pi }{180}\right),t_{03}=k\left(x_{0}-mn_{x}\right)+\left(n_{z}y_{0}-n_{y}z_{0}\right)\sin\left(\frac{\pi }{180}\right)\\ t_{10}=kn_{x}n_{y}+n_{z}\sin\left(\frac{\pi }{180}\right),t_{11}=kn_{y}^{2}+\cos\left(\frac{\pi }{180}\right),\\ t_{12}=kn_{y}n_{z}-n_{x}\sin\left(\frac{\pi }{180}\right),t_{13}=k\left(y_{0}-mn_{y}\right)+\left(n_{x}z_{0}-n_{z}x_{0}\right)\sin\left(\frac{\pi }{180}\right)\\ t_{20}=kn_{x}n_{z}-n_{y}\sin\left(\frac{\pi }{180}\right),t_{21}=kn_{y}n_{z}+n_{x}\sin\left(\frac{\pi }{180}\right),\\ t_{22}=kn_{2}^{2}+\cos\left(\frac{\pi }{180}\right),t_{23}=k\left(z_{0}-mn_{z}\right)+\left(n_{y}x_{0}\right)-n_{x}y_{o})\sin\left(\frac{\pi }{180}\right)\\ t_{30}=0,t_{31}=0,t_{32}=0,t_{33}=1 \end{array}$$

So the rotation matrix for a rotation angle of 1° is:

(8) $$\begin{array}{c} T\left(1\right)=\left[\begin{array}{cccc} t_{00} & t_{01} & t_{02} & t_{03}\\ t_{10} & t_{11} & t_{12} & t_{13}\\ t_{20} & t_{21} & t_{22} & t_{23}\\ t_{30} & t_{31} & t_{32} & t_{33} \end{array}\right] \end{array}$$

From this iteration, the rotation matrix of rotation *n*° is obtained:

(9) $$\begin{array}{c} T\left(n\right)=T{\left(1\right)}^{n}={\left[\begin{array}{cccc} t_{00} & t_{01} & t_{02} & t_{03}\\ t_{10} & t_{11} & t_{12} & t_{13}\\ t_{20} & t_{21} & t_{22} & t_{23}\\ t_{30} & t_{31} & t_{32} & t_{33} \end{array}\right]}^{n} \end{array}$$

### 2.3. Denoising Algorithm Based on Rotating Platform Distance

Point cloud noise is generated in the process of point cloud data acquisition, point cloud registration, and stereo-matching reconstruction. Due to the different absorption and reflection characteristics of light, such as the roughness, material properties, and geometric complexity of the measured object’s surface, the reflected signal is poor, and the noise point appears. When the object under test is scanned on the rotating platform, background noise may be generated, resulting in noise points generated by non-measured targets. At the same time, since the measured object is placed on the rotating platform, the camera cannot distinguish the measured object from the rotating platform. As a result, the measured object also reconstructs the rotating platform during the reconstruction, which affects the reconstruction effect and, ultimately, the measurement accuracy. Therefore, removing the noise points outside the rotating platform is necessary. After obtaining the rotating platform’s rotation axis equation and rotation matrix, point cloud reconstruction is carried out through the object pictures taken, and the noise removal is mainly carried out by Formula (10).

(10) $$\begin{array}{c} dis1=\frac{ax_{i}+by_{i}+cz_{i}+d}{\sqrt[{}]{a^{2}+b^{2}+c^{2}}} \end{array}$$

where a,b,c,d is the plane parameter of the rotating platform and $\left(x_{i},y_{i},z_{i}\right)$ is the coordinate of the point cloud data in the space. By setting the threshold of the rotating platform, the point cloud noise below and outside the rotating platform can be removed, and only the model point cloud data above the rotating platform can be retained.

Suppose the coordinate of the point cloud in space is $\left(x_{i},y_{i},z_{i}\right)$, the vertical coordinate of the point on the rotation axis is $\left(x_{d},y_{d},z_{d}\right)$, the coordinate of any point on the rotation axis is $\left(x_{1},y_{1},z_{1}\right)$, and the direction vector of the rotation axis is $\left(n_{x},n_{y},n_{z}\right)$, then:

(11) $$\left\{\begin{array}{c} xd=n_{x}t+xl\\ yd=n_{y}t+yl\\ zd=n_{z}t+zl \end{array}\right.$$

Since the dot product of the vector $\left(x_{i}-xd,y_{i}-yd,z_{i}-zd\right)$ and the direction vector of the line $\left(n_{x},n_{y},n_{z}\right)$ is 0, then:

(12) $$\begin{array}{c} n_{x}\left(x_{i}-xd\right)+n_{y}\left(y_{i}-yd\right)+n_{z}\left(z_{i}-zd\right)=0 \end{array}$$

In connection with (11) and (12), the calculated parameter *t* is:

(13) $$\begin{array}{c} t=\frac{\left(n_{x}\left(x_{i}-xl\right)+n_{y}\left(y_{i}-yl\right)+n_{z}\left(z_{i}-zl\right)\right)}{\left(n_{x}^{2}+n_{y}^{2}+n_{z}^{2}\right)} \end{array}$$

Then the distance from the point to the line can be calculated, and the threshold of noise removal can be carried out from this distance.

(14) $$\begin{array}{c} dis2=\sqrt[{}]{{\left(x_{i}-xd\right)}^{2}+{\left(y_{i}-yd\right)}^{2}+{\left(z_{i}-zd\right)}^{2}} \end{array}$$

**Figure 3:**
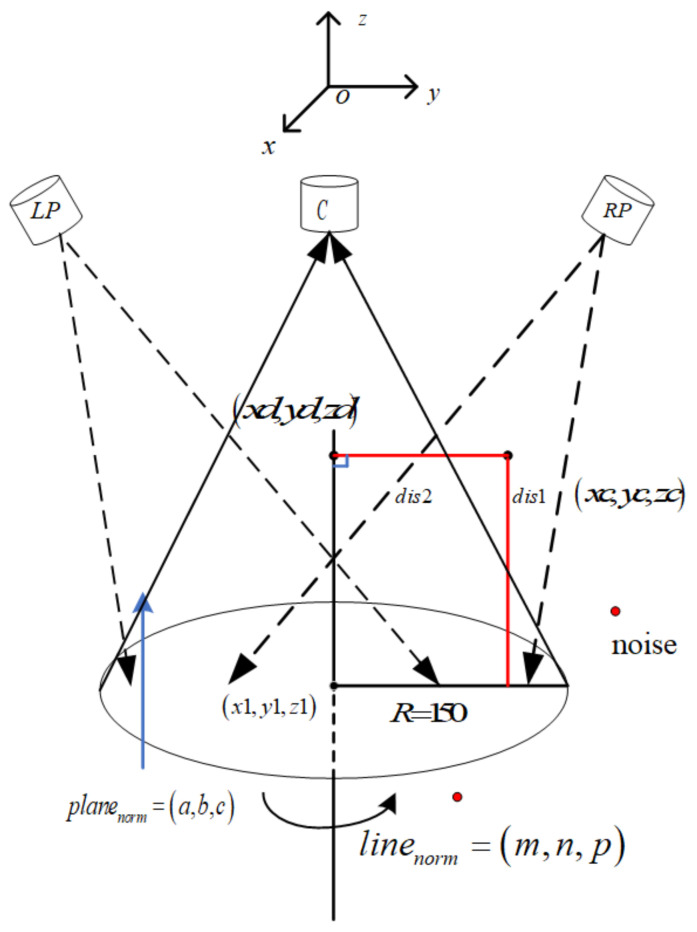
Schematic diagram of rotating platform denoising.

A single perspective point cloud data model with noise removed can be obtained. The noise outside the rotating platform can be removed by setting a threshold $\left(150-dis2\right)$ < 0.01 combined with the radius of the rotating platform, and only the point cloud data model inside the rotating platform space can be retained. The details can be seen in Figure 3.

### 2.4. Multi-View Point Cloud Registration Based on Rotating Platform

Multi-view point cloud data acquisition and 3D reconstruction based on the rotating platform can quickly obtain the surface information of objects. By rotating the platform at a certain angle, the surface of the measured object is gradually moved into the field of view of the camera, Thus, the three-dimensional information of the surface of the measured object under different perspectives can be obtained. Then the camera coordinates system, and the rotating table coordinate system data were unified into the same coordinate system through camera and rotation axis calibration, and 3D reconstruction was realized. Assume that the point cloud data model obtained from each perspective is Xi, where i=1,2…n, is the angle of view. In this paper, the 0° point cloud data is taken as the benchmark, and the angle of rotation of 60° is taken as an example. The rotation matrix obtained by the other 5 Angle point clouds is used for rough registration of the point clouds, namely:

(15) $$\begin{array}{cc} \; & X_{0}\\ \; & X_{60}^{\prime }=X_{60}T\left(60\right)\\ \; & X_{120}^{\prime }=X_{120}T\left(120\right)\\ \; & X_{180}^{\prime }=X_{180}T\left(180\right)\\ \; & X_{240}^{\prime }=X_{240}T\left(240\right)\\ \; & X_{300}^{\prime }=X_{300}T\left(300\right) \end{array}$$

where $X_{i}$ is the point cloud data from the original perspective and $X_{i}^{\prime }$ is the point cloud data at the angle of 0° after $X_{i}$ is rotated by i degree. At this time, all point cloud data models are located in the same perspective, and the rough matching process is completed. Finally, the point cloud precision matching is carried out by the latest iteration registration algorithm to obtain the completed point cloud data model.

## 3. Experiment

### 3.1. Evaluation of Uncertainty

**Figure 4:**
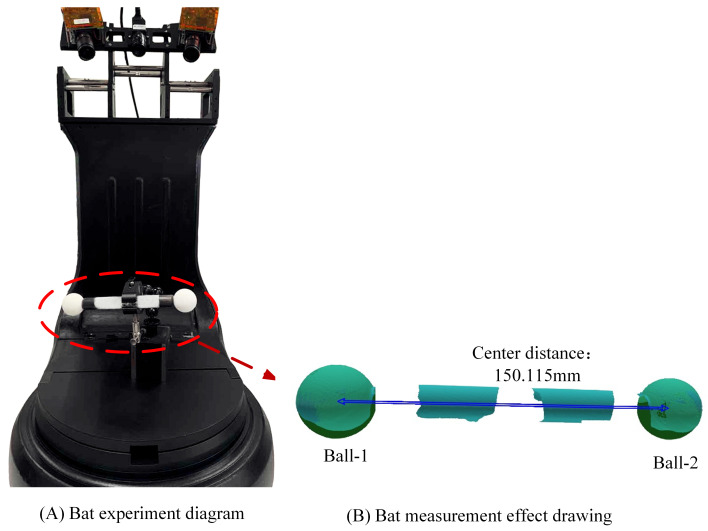
An experimental rendering of the bat.

In order to verify the uncertainty of the proposed method, a standard ball bat made of ceramic material is used as the workpiece to be evaluated, As shown in Figure 4. The reference value of the center distance of the ball is 150.1164 mm, the average diameter of the ceramic ball 1 is 30.0008 mm, and the average diameter of the ceramic ball 2 is 30.0015 mm. The center distance of the ball was measured at 150.1147 mm by a Hexagon coordinate machine. The measured data are as follows.

Based on the measurement principle and method [32], the measurement model is obtained:

(16) $$\begin{array}{c} SD=MAX\left(\left|SD_{i}\right|\right)=MAX\left(\left|L_{ai}-L_{r}\right|\right) \end{array}$$

where $L_{ai}$ is the measurement value of spherical center distance at the position *i* and $L_{r}$ is the reference value of the center distance of the ball.

The measurement uncertainty of the bat reference value is 5.0 μm, including factors *k* = 2, and then the component of the standard uncertainty introduced by the bat reference value $u_{1}\left(L_{r}\right)=2.5$ μm.

The linear expansion coefficient of the bat is $\left(8\pm 1\right)\times 10^{-6}$ °C${}^{-1}$, the length is 150.1164 mm, and the temperature measurement error is better than 0.5 °C. If it is processed according to a uniform distribution, then $u_{2}\left(L_{r}\right)=\frac{L\alpha \Delta t}{\sqrt{3}}\mu$m.

The linear expansion coefficient of the bat is uniformly distributed within the half-width interval $1\times 10^{-6}$ °C${}^{-1}$, and the ambient temperature in the laboratory is estimated according to the average deviation 5 °C, then:

$$u_{3}\left(L_{r}\right)=\frac{L(t-20)\Delta \alpha }{\sqrt[{}]{6}}$$

The standard uncertainty introduced into each component of the synthetic standard instrument:

$$u_{c}=u\left(L_{r}\right)=\sqrt[{}]{u_{1}^{2}\left(L_{r}\right)+u_{2}^{2}\left(L_{r}\right)+u_{3}^{2}\left(L_{r}\right)}$$

According to the measured data in Table 1, the class A standard uncertainty can be obtained as follows:

$$u\left(L_{ai}\right)=\sqrt[{}]{\frac{\sum_{i=1}^{7}{\left(s_{i}-\bar{s}\right)}^{2}}{7\times 6}}$$

where $s_{i}$ is the measurement value of the *i*-th position, and $\bar{s}$ is the arithmetic mean value of the measured data.

Because each uncertainty component is not correlated with the other, the synthetic standard uncertainty can be obtained according to the uncertainty propagation rate:

$$u_{c}\left(SD\right)=\sqrt[{}]{u^{2}\left(L_{ai}\right)+u^{2}\left(L_{r}\right)}$$

It can be seen from the above calculation that the measurement uncertainty of the instrument is 7.938 μm, which better explains the deviation between the measurement result and the true value, and proves the accuracy of the measurement.

### 3.2. Experimental Equipment and Process

#### 3.2.1. Experimental Equipment

**Figure 5:**
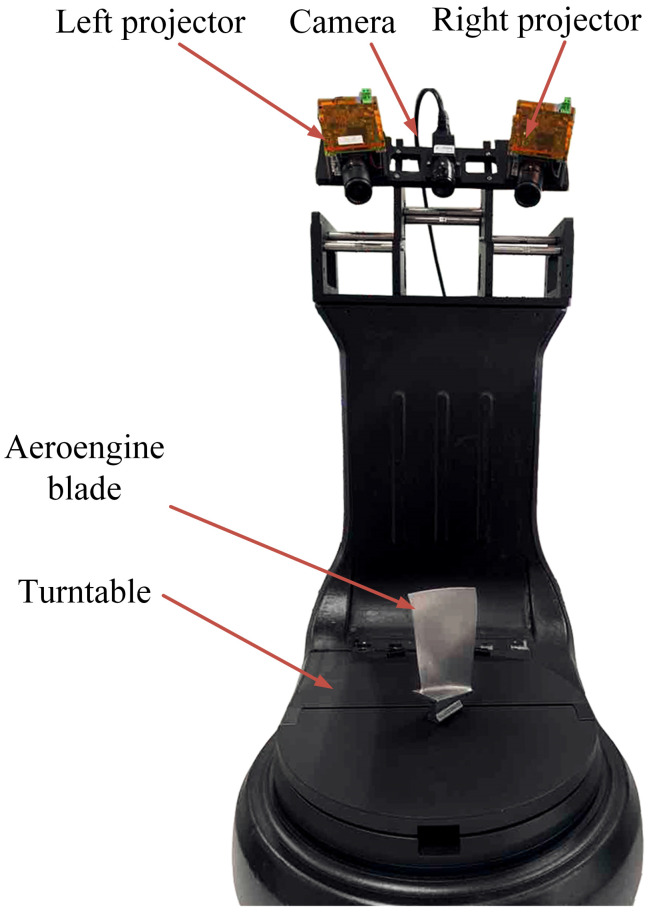
Three-dimensional scanning equipment.

In order to achieve the high-precision 3D reconstruction of the measured object, the relative position between the structured light system and the rotating platform is kept unchanged, and the center of the rotation axis is kept unchanged. The rotating platform is used to obtain point cloud data from different perspectives. In the experiment, the left and right projectors combined with a camera in the middle were used to form a structural light measurement system. The structural light system formed a 27° angle with the rotation axis of the rotating platform. The experimental device is shown in Figure 5. The rotating platform rotates every 60°, and the measured object is sent into the camera’s field of view. The camera records the deformation fringe, calculates the model mapping relationship between the rotating platform’s rotation angle and the point cloud’s rigid transformation, and completes the system calibration. According to the established model mapping relationship, the point cloud of the workpiece from multiple perspectives can be unified into the same coordinate system to complete the rough registration of the point cloud. Finally, the closest point iteration method is used for precise registration. The structure light measurement system uses a DLP4500SL projector with a resolution is 1140 × 912; Vision manufactures industrial camera HS510GC with resolution of 2464 × 2056.

**Figure 6:**
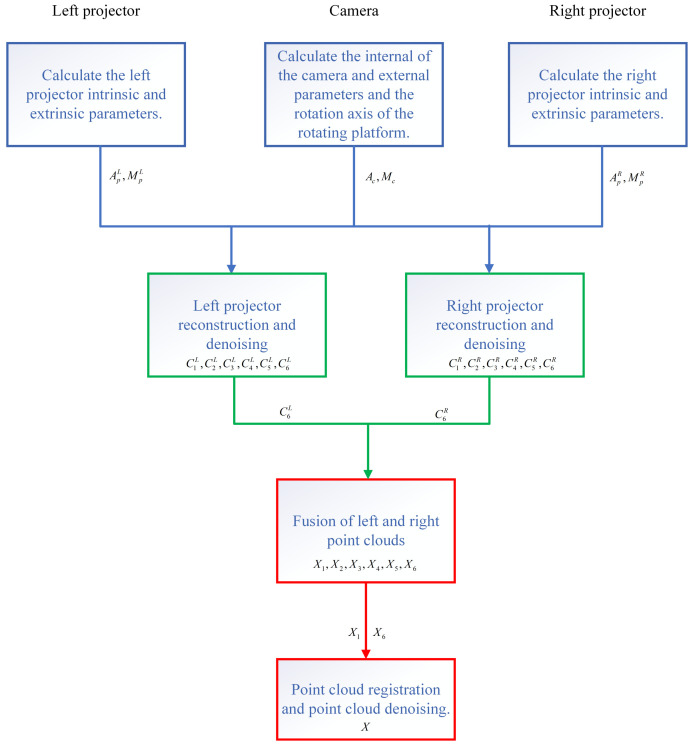
The flow chart of the proposed method.

As shown in Figure 6, our approach can be divided into four steps, each represented by a different color. Firstly, the internal and external parameters of the left and right projections are calculated. The internal and external parameters of the camera are calculated, and the rotation axis of the rotating platform is determined. Then the camera is used to capture the modulated Gray code and phase-shift fringe of the object surface, and the point cloud is denoised under the left and right projections. The parallax map of the object under the same measurement angle can be obtained. In the third step, a parallax map is used for phase error correction, and point cloud data fusion is carried out to fill the point cloud holes caused by edge occlusion. In the last step, the point cloud registration and the second denoising of the point cloud are carried out, followed by the three-dimensional reconstruction of the object to be measured.

**Figure 7:**
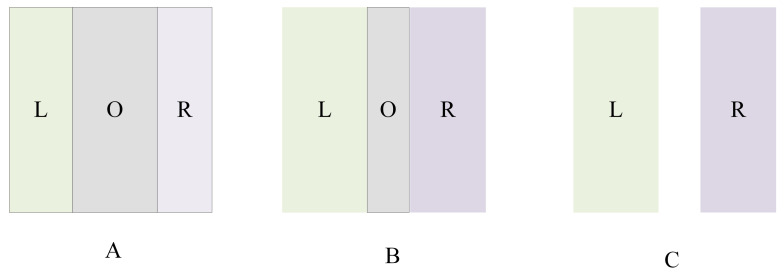
Diagram of the overlapping areas at different rotation angles. (**A**) indicates that when the rotation angle is 30°, its overlap area is larger; (**B**) indicates that when the rotation angle is 60°, the overlap area has been reduced compared to 30°; (**C**) indicates there is no overlap area.

**Figure 8:**
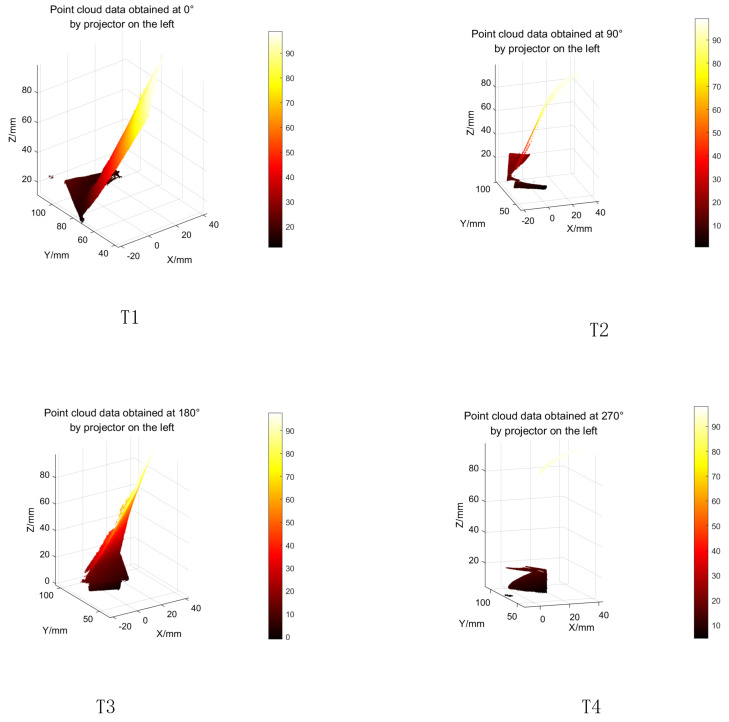
Point cloud data of the aero engine blade at a rotation angle of 90°; T1 is the point cloud data plot with a rotation angle of 0°, T2 is the point cloud data plot with a rotation angle of 90°, T3 is the point cloud data plot with a rotation angle of 180°, and T4 is the point cloud data plot with a rotation angle of 270°.

We set the rotation angle to 60° to optimize the acquisition of point cloud data, make the amount of point cloud data moderate, facilitate the later point cloud processing, and avoid the loss or redundancy of point cloud data, thus ensuring the accuracy and speed of the 3D reconstruction. The principle of angle selection is that it needs to be divisible by 360°. When using smaller rotation angles, such as 20°, 30°, 45°, more image groups need to be captured during a full 360° rotation, which takes more time than a 60° rotation angle. Secondly, there are a large number of overlapping areas in camera shooting in the process of small-angle rotation, resulting in the redundancy of phase extraction and point cloud data angle. In the process of 3D reconstruction, these redundant point cloud data must be removed, resulting in a large amount of calculation. When the rotation angle is set to 90°, due to the limited imaging field of view of the camera, some areas of the measured object will fail to enter the camera field of view. As a result, the measured object cannot be fully imaged, point cloud data is missing, and effective splicing of point cloud data cannot be realized, resulting in reconstruction failure. In summary, it is more appropriate to set the rotation angle to 60°. As shown in Figure 7, L represents the area projected on the left, R represents the area projected on the right, and O represents the overlapping area. Subfigure A indicates that when the rotation angle is 30°, its overlap area is larger; subfigure B indicates that when the rotation angle is 60°, the overlap area has been reduced compared to 30°. When the rotation angle is 90°, there is no overlap area. For a rotation angle of 90°, we also carried out relevant experiments, and the specific results are shown in the figure below. As can be seen from Figure 8. T4, due to the large rotation angle, the image will be missing and cannot be completely fused.

We used the aero engine blade to conduct experiments. The experimental results of using a 90° rotation angle are shown in Figure 8. Due to the large rotation angle, the image is missing and cannot be completely fused.

#### 3.2.2. Distance-Based Point Cloud Background Noise Removal

We keep the equipment in the structured light system fixed, place the object to be measured on the rotating platform, and the rotating platform then drives the object to rotate at a certain speed. The camera collects the point cloud data from 6 different perspectives. When the object to be measured is at 0°, the projector on the right projected a nine-order Gray code combined with eight-step phase shift and one black and white image to the surface of the object that is to be measured. The camera collected the modulated fringe patterns on the object’s surface and carried out Gray code decoding and phase shift decoding, respectively. At the same time, background noise removal was done on the point cloud data under different perspectives based on the distance algorithm. In addition, the left projector also needs to project the designed structured light image onto the object to be tested then decode and reconstruct it in three dimensions, respectively.

#### 3.2.3. Multi-View 3D Data Fusion

The rotating platform obtains the point cloud data from different perspectives to realize the comprehensive reconstruction of the object to be measured. When the right projector projects structural light onto the object to be measured, the camera will obtain the point cloud data on the right side of the object to be measured. Similarly, when the left projector projects structural light onto the object to be measured, the camera will also obtain the point cloud data on the left side of the object to be measured. Under the same rotation angle, the structural light projected by the left and right projectors overlaps partially on the object to be measured, so the point cloud data will also overlap partially. In order to further ensure the accuracy and reconstruction effect of point cloud data fusion, the intersection processing of two-point cloud data is carried out after the point cloud data is unified into the same coordinate system, and the overlapping point cloud data is retained and triangulated.

#### 3.2.4. Reconstruction Experiment

The aero-engine blade profile is a free-form surface with a large size span, and its geometry and size have a decisive effect on its performance. The quality inspection of an aero-engine profile is mainly to detect the geometry of the blade profile. The traditional CMM method measures each measuring point on the blade profile and calculates the measured blade’s geometrical size and shape error. This method has high measurement costs and low efficiency. The multi-viewpoint cloud registration method can be used for rapid reconstruction and precision measurement of aero engine blades.

**Figure 9:**
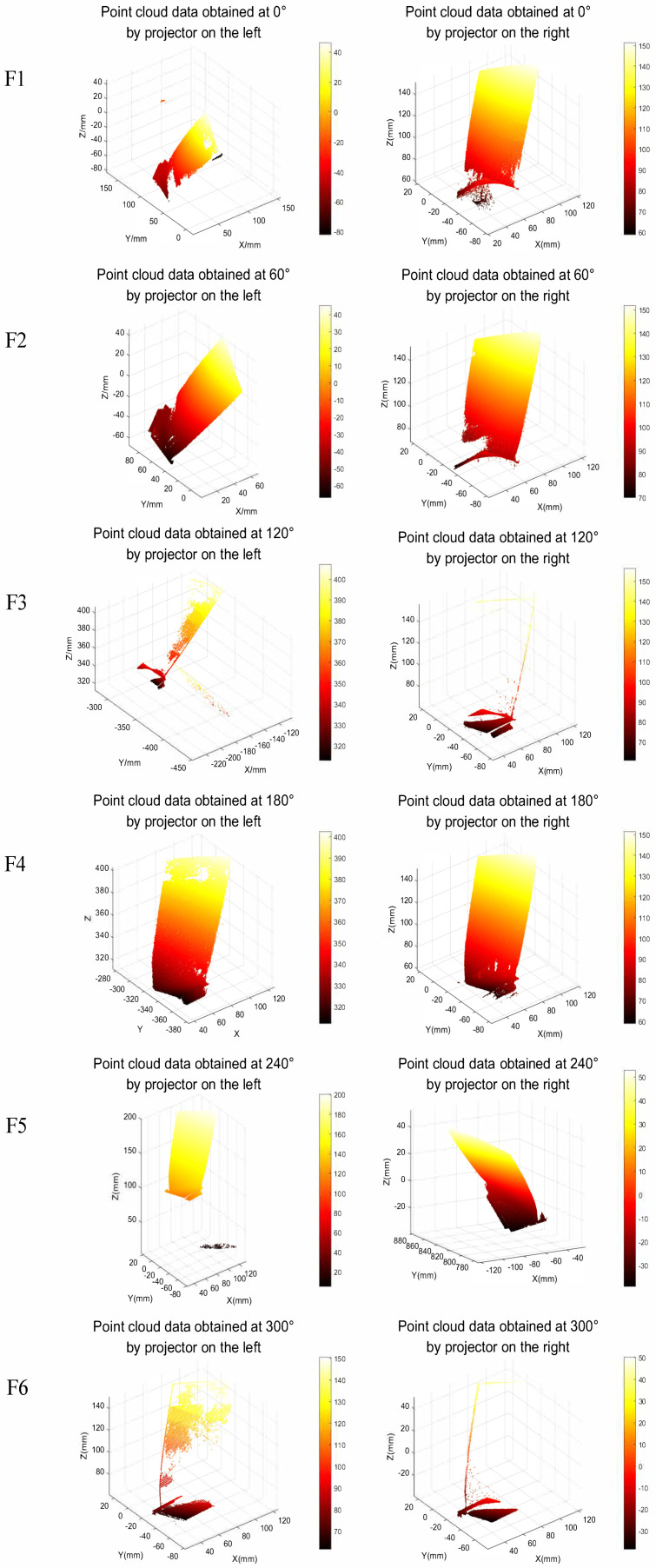
Multi-view point cloud data fusion, the blade on the rotating platform at 60° counterclockwise rotation. F1, F2,…F6 indicates 0°, 60°…At 300°, the left and right projectors capture the point cloud data obtained by the aviation blades.

**Figure 10:**
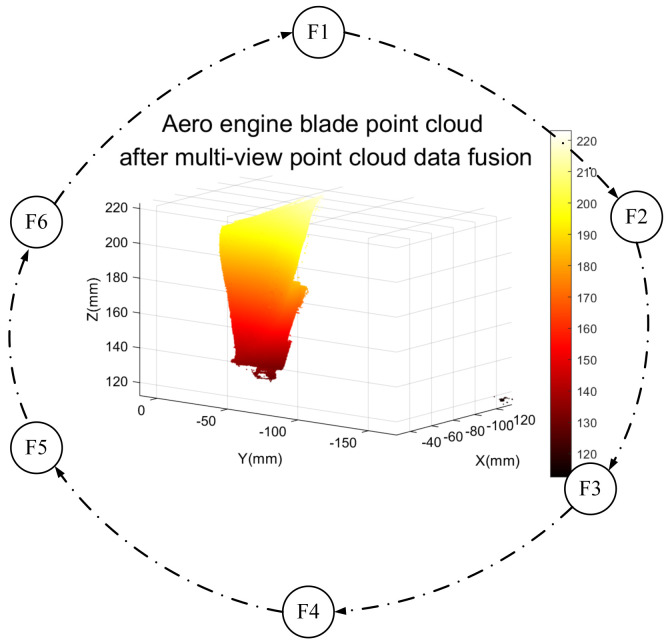
Aviation blade model after point cloud fusion.

Figure 9 shows the registration process of point cloud data fusion from multiple perspectives. At an angle of 0°, the left and right point cloud data are obtained, respectively. According to Figure 6, the left and right point cloud fusion realizes the intersection of point cloud data to extract the public area. In the process of fusion registration, the left and right point clouds will complement each other to fill the holes in the point cloud. Then, the left and right point cloud data were obtained at 60°, 120°, 180°, 240°, and 300°, respectively, for intersection point cloud fusion, and the multi-view point cloud data under each angle was further fused into a complete three-dimensional model. The aviation blade model after point cloud fusion is shown in Figure 10. The basis of multi-view point cloud data fusion is actually based on the imaging of each single view camera. Figure 9 illustrates this in detail.

**Figure 11:**
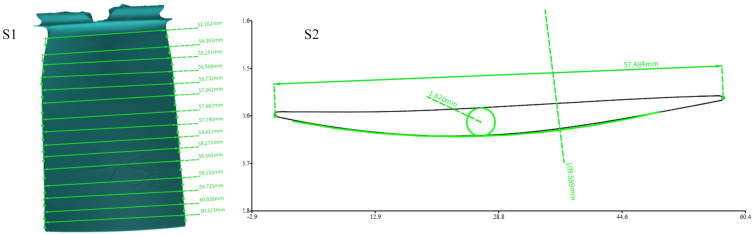
S1 is the measurement of the aero engine blade diameter; S2 is the measurement of the blade section size.

**Figure 12:**
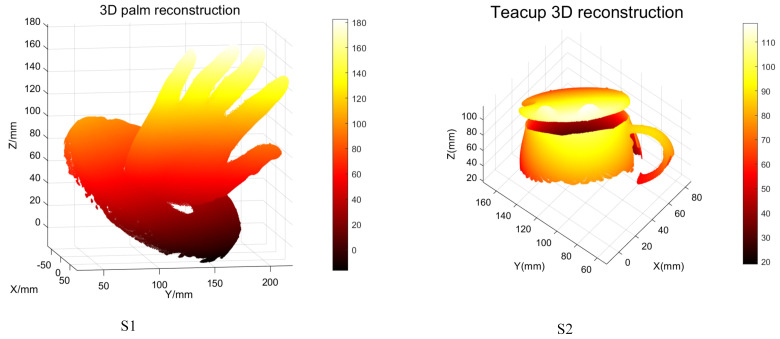
S1 is the three-dimensional reconstruction of the plaster model of the palm; S2 is the 3D reconstruction model of the teacup.

The dimension measurement of the aero engine blade is very important in its production and maintenance. Due to the complexity of the blade shape and the large size fluctuation, the measurement of the aero-engine blade mainly includes the blade shape, the position of the front and rear edges, the blade length, and the blade torsional surface. Diameter and section measurements were carried out for the reconstructed blades, as shown in Figure 11. In addition, other relevant 3D reconstruction experiments were further supplemented, as shown in Figure 12.

## 4. Discussion

The process of noise removal of multi-viewpoint cloud registration aims to maintain the target characteristics while eliminating noise. To achieve this, the object to be measured is placed on a rotating platform and rotated at a certain angle. This rotation allows different parts of the object’s surface to sequentially enter the camera’s field of view, thus obtaining the three-dimensional point cloud information of the object surface from various angles. However, this approach has the following limitations: It is limited by the ray casting angle and camera field of view of DLP; therefore, the size of the measured object is constrained, especially when there are protruding parts of complex components, DLP light will be blocked, resulting in a large point cloud void. Additionally, the ambient light will increase the point cloud noise, resulting in increased difficulty in denoising, especially in the strong light environment, which will lead to strong reflections on the measured object. The algorithm’s robustness is poor, thus affecting the final measurement accuracy. Furthermore, due to the influence of surface characteristics of objects with high dynamic range, it is difficult to fuse point clouds in multi-view point cloud registration due to overexposure.

## 5. Conclusions

Some advantages in this paper include: (1) when the structured light projection system with two projectors combined with a single camera is used, point cloud data holes caused by the shadow region of the measured object’s structure can be effectively avoided; (2) three-dimensional reconstruction of the measured object can be realized in the whole process without manual intervention, and the point cloud data can be spliced and fusion automatically, with higher overall accuracy and higher degree of automation; (3) it shows that the aero engine blade is measured more efficiently than that measured by the coordinate machine.

In this paper, a structured light projection system with two projectors and a single camera is proposed based on the Gray code combined with the phase shift method, and a rotating platform is used to reconstruct the object under multiple perspectives in order to reduce the shadow measurement area and enhance the measurement ability of the system. A distance-based point cloud noise removal algorithm was proposed to optimize the platform noise and realize the point cloud data fusion under multiple perspectives. The point convergence scanned under each perspective was converted into the same coordinate system, and the three-dimensional reconstruction of the top of complex objects and some areas blocked by light was effectively realized. The experimental results show that the system can improve the robustness of the system, and the synthesis standard is uncertain 7.938 μm. 

## Figures and Tables

**Table 1:** Measured 150 mm ball center distance under different positions and poses.

Position	Pose 1	Pose 2	Pose 3	Pose 4	Pose 5	Pose 6	Pose 7
Distance * (mm)	150.155	150.169	150.141	150.110	150.122	150.139	150.130

* Measured Distance between the center of the sphere.

## Data Availability

The data is unavailable due to privacy or ethical restrictions.
